# Breast Cancer Pathology, Receptor Status, and Patterns of Metastasis in a Rural Appalachian Population

**DOI:** 10.1155/2014/170634

**Published:** 2014-01-09

**Authors:** Linda Vona-Davis, David P. Rose, Vijaya Gadiyaram, Barbara Ducatman, Gerald Hobbs, Hannah Hazard, Sobha Kurian, Jame Abraham

**Affiliations:** ^1^Department of Surgery, Mary Babb Randolph Cancer Center, Robert C. Byrd Health Sciences Center, West Virginia University, P.O. Box 9238, Morgantown, WV 26506, USA; ^2^Breast Cancer Research Program, Mary Babb Randolph Cancer Center, West Virginia University, Morgantown, WV 26506, USA; ^3^West Virginia University, Morgantown, WV 26506, USA; ^4^Department of Pathology, West Virginia University, Morgantown, WV 26506, USA; ^5^Department of Statistics and Community Medicine, West Virginia University, Morgantown, WV 26506, USA

## Abstract

Breast cancer patients in rural Appalachia have a high prevalence of obesity and poverty, together with more triple-negative phenotypes. We reviewed clinical records for tumor receptor status and time to distant metastasis. Body mass index, tumor size, grade, nodal status, and receptor status were related to metastatic patterns. For 687 patients, 13.8% developed metastases to bone (*n* = 42) or visceral sites (*n* = 53). Metastases to viscera occurred within five years, a latent period which was shorter than that for bone (*P* = 0.042). More women with visceral metastasis presented with grade 3 tumors compared with the bone and nonmetastatic groups (*P* = 0.0002). There were 135/574 women (23.5%) with triple-negative breast cancer, who presented with lymph node involvement and visceral metastases (68.2% versus 24.3%; *P* = 0.033). Triple-negative tumors that metastasized to visceral sites were larger (*P* = 0.007). Developing a visceral metastasis within 10 years was higher among women with triple-negative tumors. Across all breast cancer receptor subtypes, the probability of remaining distant metastasis-free was greater for brain and liver than for lung. The excess risk of metastatic spread to visceral organs in triple-negative breast cancers, even in the absence of positive nodes, was combined with the burden of larger and more advanced tumors.

## 1. Introduction

Despite the progress that has been made in the diagnosis and treatment of early stage breast cancer, a substantial proportion of patients still go on to develop incurable distant metastatic disease. The lack of estrogen receptor (ER) and progesterone receptor (PR) expression in breast cancer is associated with an increased likelihood of visceral metastases and a particularly poor prognosis [1–4]. So-called triple-negative breast cancers lack both ER and PR and also human epidermal growth factor 2 receptor (HER2) expression. This phenotype is particularly common in younger women [5–7] and is likely to be accompanied by distant, hematogenous metastases that usually occur in the first five years after the initial diagnosis and are associated with relatively short relapse-free and overall survival times [6, 8, 9]. Both steroid hormone receptor-negative breast cancers [10–12] and triple-negative tumors [5, 12, 13] are more common in women with a socioeconomically deprived background.

Bone is the most commonly observed site for distant metastases and is the location of 30–40% of first tumor recurrence [14, 15]. Women with their first recurrence occurring in the skeleton have a better prognosis than those with visceral metastases to the liver, lung, or brain [15, 16]. Bone metastases occur particularly with ER-positive tumors and, in contrast to the early presentation of visceral metastases, at any time over a 10–15 year period after the initial surgical treatment [17].

Obesity, which is more prevalent in women with low income and educational levels [18], has been related to more advanced breast cancer at the time of diagnosis and a poor prognosis in both premenopausal and postmenopausal women [1]. It is also associated with the triple-negative phenotype [13, 19]. Several studies found that obese women were at an increased risk for breast cancer metastasis to distant sites (reviewed in [3]), and Dawood et al. [20] have reported that overweight and obese women with locally advanced breast cancer, regardless of their menopausal status, are at a greater risk for visceral metastases than their lean counterparts.

West Virginia, the only state that is entirely in Appalachia, has a population that is 95% White, and in the years of this study, it ranked sixth highest among the states for the percent of the population that was living below the poverty line [21] and was fourth for the prevalence of obesity [22]. Although it has one of the lower incidence rates for breast cancer among the 50 states, it ranked sixteenth in mortality rate for the disease [23]. In a retrospective study in West Virginia of 620 White patients with invasive breast cancer, we found that triple-negative breast cancers comprised an unusually high proportion of the various combinations of ER, PR, and HER2 receptors, which occurred particularly in younger women and in association with obesity and larger primary tumors at the time of diagnosis [13].

The purpose of the present study was to examine the relationships between tumor steroid hormone receptor and HER2 status and patient adiposity and the development of distant metastases, in this same cohort of breast cancer patients. Given the relatively poor outcomes for triple-negative disease, we compared the proportions of breast cancer patients who develop metastases at various sites compared with other types of breast cancer.

## 2. Patients and Methods

### 2.1. Study Population

Women with breast cancer treated according to clinical protocols, conducted in the Breast Care Clinic of the Mary Babb Randolph Cancer Center, composed the study group for this West Virginia University Institutional Review Board-approved investigation. For followup, the patients were seen every three months for clinical examination, chest X-ray, and evaluation for the tumor marker CA 27.29; imaging studies were performed if they were considered at high risk of recurrence. After the first 2 years, the patients were seen every six months for clinical breast examination and tumor marker studies. Medical records and pathology reports on 712 breast cancer patients seen in the clinic were reviewed and those with disease limited to carcinoma *in situ* excluded from the study, as were 6 patients who had developed carcinoma of the lung subsequent to the diagnosis of breast cancer.

An age cutoff at 50 years was used as a surrogate for defining menopausal status [13]. The body mass index (BMI), tumor size, histologic grade, and regional lymph node involvement were determined as described previously [13]. The BMI values were categorized according to WHO criteria: normal or underweight (lean), <25.0 kg/m^2^; overweight, 25.0 to 29.9 kg/m^2^; obese, 30.0 kg/m^2^ or higher.

Distant metastasis-free survival was defined as the interval between the initial diagnosis and the time at which a metastasis to bone and/or a visceral site (liver, lung, brain) was first detected. If more than one location was involved, the designation of first metastatic site was prioritized in the order: liver, lung, brain, and bone.

### 2.2. Pathologic Evaluation

These procedures were described in detail earlier [13]. Briefly, ER, PR, and HER2 expression were determined on paraffin-embedded tumors by immunohistological staining using DAKO antibodies. HER2 status was assessed in the hospital cytogenetics laboratory using the CBII monoclonal antibody from Ventana Medical Systems and ChromaVision image analysis. HER2 positivity was defined as strong complete membrane staining in at least 10% of the tumor cells and scored as 3+. Scores of 0 and 1 were classed as negative and the positivity of a 2+ rating was affirmed by fluorescence *in situ* hybridization. Tumors were classified as “triple-negative” if they were negative for ER, PR, and HER2/neu.

### 2.3. Statistical Analysis

Analyses of demographic information and tumor characteristics were compared between metastasis-free women and those with either kind of distant metastases using a *t*-test. For comparison of categorical variables a chi-square statistic was used. Comparisons between more than two groups were performed with ANOVA. A Wilcoxon rank sum test was used for certain variables. The principal subgroups of interest were age (<50 versus ≥50 years), BMI (<30 versus ≥30 kg/m^2^), tumor size, grade, and lymph node status. Two-tailed tests were used at all times, and statistical significance was set *a priori* at *P* < 0.05. Means are reported with standard errors (±SE). Rates of recurrence were evaluated using the date of diagnosis and the date of first metastatic event in months to either bone or visceral sites. Some observations were deleted if distant metastases were detected at the time of breast cancer diagnosis. The log-rank test was used to examine the statistical significance observed for recurrence-free survival time between triple-negative and other subgroups. We used Pearson chi-square analysis to assess differences between groups for 10-year recurrence-free survival. In addition, a multiple logistic regression was run with metastasis as the response including possible confounding variables such as age, BMI, receptor status, and tumor size in the model. All statistical analyses used the Statistical Analysis System software program (JMP/Pro version 10; SAS Institute, Cary, NC).

## 3. Results

### 3.1. Patient Age and Distant Metastases

A group of 687 patients with invasive breast cancer was included in the study (Table 1). The median follow-up period was 4.7 years with a range of 0 to 10.3 years. Ninety-five of the 687 (13.8%) breast cancer patients developed distant metastases to bone (*n* = 42) and visceral (*n* = 53) sites during the study period. Of the patients we followed, 493 (71.8%) were 50 years or older and 194 (28.2%) were younger. The mean age at the time of diagnosis of the patients who remained free of distant metastases was 58.7 years, and for those with bone or visceral metastases it was 53.6 and 56.0 years, respectively. Women were more likely to experience a disease recurrence at age 50 or younger, 17/194 (8.8%) and 21/194 (10.8%), for distant bone and visceral metastasis, compared to 25/493 (5.1%) and 32/493 (6.5%) in women older than 50 years of age (*P* = 0.028).

In 42 of the 95 patients (44.2%) the first distant metastasis was to bone (Table 2). Of the 53 women whose first metastasis was to the viscera, the organ most often involved was the liver (39.6%), followed by the lung (37.7%), brain (20.8%), and ovary (1.9%). We compared the disease free interval for bone and combined visceral sites using the log-rank test. The median disease free interval for bone was 19.9 months compared to 13.9 months for viscera, showing a trend to shorter recurrences visceral sites (*P* = 0.079). Among those who developed visceral metastases to liver, lung, and brain (excluding the single ovary case), the median time to recurrence was the shortest in lung, 9.6 months, compared to 20.9 months in liver and 25.8 months for brain (*P* = 0.024). Triple-negative patients with metastasis to lung had a median survival in months of 18.0 (range 5.6–24.4), followed by brain 26.9 (range 13.1–27.6), liver 39.0 (range 24.1–40.0), and bone 50.9 (range 38.0–60.6) months when compared to other breast cancer subtypes (*P* = 0.017).

**Figure 1:**
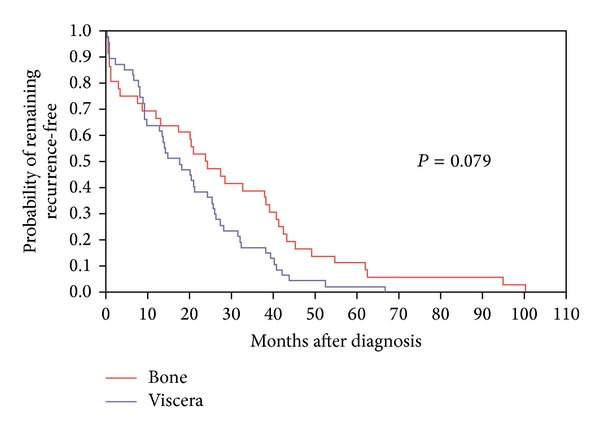
Metastatic-free interval for distant bone and visceral (liver, lung, and brain) metastases.

Kaplan-Meier curves comparing the appearance of distant metastasis to bone and visceral sites over time are shown in Figure 1. Observations were deleted if distant metastases were detected at the time of the original diagnosis. Metastases to liver, lung, and brain (combined) occur more rapidly and are virtually complete in the first 5 years of followup, compared with the diagnosis of bone metastases which occurred more gradually over time (*P* = 0.079). When plots for the individual visceral sites were compared, the probability of remaining distant metastasis-free was greater for brain and liver than for lung and the median time for recurrence was less than one year after the initial diagnosis of breast cancer (*P* = 0.024).

### 3.2. Tumor Size, Grade, Nodal Status, and Distant Metastases

In cases where tumor size was available, the mean diameter was 2.2 ± 0.8 cm for 556 tumors without detected distant metastasis and for 79 tumors that had undergone metastasis to distant sites (bone and viscera combined) was 3.9 ± 0.3 cm (Table 1; *P* < 0.0001). In addition, there were significantly fewer T1 tumors (<2.0 cm) in the women who developed distant metastases (*P* < 0.0001). The apparently greater mean maximum diameter of 45 tumors that had metastasized to a visceral site (4.1 ± 0.3 cm) compared with 34 that had metastasized to bone (3.6 ± 0.3 cm) and the difference in the frequency of T1 tumors for the two metastatic groups were not statistically significant (*P* = 0.561 and 0.435, resp.).

Distant metastasis, and particularly that to visceral sites, was associated with higher tumor grade. Although the numbers of patients with distant metastases were small (Table 1), 80.0% of tumors that subsequently metastasized to liver, lung, or brain, compared with 67.5% of those associated with bone metastases, were grade 3. Also, 51.7% of 513 primary tumors that had not undergone distant metastasis were classified as grade 3. Significantly more women with visceral metastasis presented with grade 3 (40/50) tumors compared to bone and nonmetastatic groups (*P* = 0.0002).

The axillary nodal status at the time of diagnosis was known for 671 of the 687 patients and 167 (24.9%) had positive nodes (Table 1). The difference between the frequency of nodal involvement in the patients without distant metastases (21.5%) and those with metastases to an osseous (39.0%) or visceral site (50.9%) was highly significant (*P* = 0.0002); that between those whose first distant metastasis was to the viscera and bone was not statistically significant (*P* = 0.249).

Despite the relatively low risk of subsequent distant metastasis associated with the absence of detected axillary lymph node involvement, 49.3% of the patients in this prognostic category had grade 3 tumors, which compared with 50.8% of the lymph node positive patients, a difference that was not significant. The development of distant metastases in lymph node negative patients was associated with a higher prevalence of grade 3 tumors, these occurring in 33 of 49 (67.3%) women compared with 205 of 391 (52.4%) of the nonmetastatic group (*P* = 0.0001).

### 3.3. Metastasis and Receptor Status

The prevalence of ER, PR, and HER2 positive breast cancers in the nonmetastatic group of patients and those with first metastasis to bone or a visceral site is shown in Table 3. Of the 488 tumors without distant metastasis during the observation period, 70.7% expressed the ER; in comparison, while the prevalence of ER-positive tumors in the patients who subsequently had osseous metastases was 78.6%, in those with metastases to visceral sites it was only 41.5% (*P* = 0.0005). Similar differences were evident in expression of the PR (Table 3).

Assays for both HER2 and the two steroid receptors had been performed on the tumors of 488 women without evidence of distant metastasis and 98 (20.8%) showed HER2 overexpression. HER2 overexpression was also present in 5 of 36 (13.9%) assayed tumors that subsequently metastasized to bone and 6 of 50 (12.0%) that were associated with visceral metastases: none of these differences were not statistically significant (Table 3; *P* = 0.245).

Of a total of 574 tumors for which we had complete receptor information, 135 were negative for all three receptors, giving an overall prevalence of triple-negative tumors of 23.5%. Triple-negative breast cancers were associated with visceral metastases. As shown in Table 3, the frequency of triple-negative breast cancers in patients with first metastases to visceral sites was approximately double that in those whose tumors had not metastasized to distant sites during the study period (*P* = 0.002) and approximately 3-fold that occurring in association with subsequent first metastasis to bone (*P* = 0.003).

The relationships between metastatic behavior and sites of distant metastasis, nodal status, tumor grade, and the triple-negative phenotype are summarized in Table 4. The triple-negative phenotype was not associated with a high prevalence of lymph node involvement in women who remained free of distant metastases (*P* = 0.610). However, there was a particularly high frequency of node-positive triple-negative breast cancer patients with visceral metastases (Table 4: 68.2% versus 24.3%; *P* = 0.033), whereas the combination of lymph node involvement and metastasis to osseous sites was not related to the triple-negative phenotype (*P* = 0.454).

Distant metastasis was associated with higher tumor grade (Table 4). There was a higher frequency of grade 3 tumors in the triple-negative group that had not undergone distant metastasis compared with other breast cancers, 74.8% and 45.5%, respectively (*P* < 0.0001). The relationship between histologic grade and bone metastasis was not modified by the triple-negative phenotype. Furthermore, although for women with visceral metastases, 90.9% of the triple-negative tumors were grade 3, compared with 71.4% of the non-triple-negative tumors, the trend did not achieve statistical significance (*P* = 0.092).

Table 5 summarizes the size of tumors from patients without distant metastases and those with first metastasis to osseous or visceral sites, in relation to their ER and triple-negative status. In the nonmetastatic group, there were similar increases in the mean maximal diameter of the ER-negative, but PR and/or HER2-expressing tumors and the triple-negative tumors (2.7 ± 0.2 and 2.8 ± 0.2 cm, resp.) compared with the ER-positive tumors (2.0 ± 0.9 cm). There was a significant association between metastatic pattern and tumor size for the three receptor subtypes. Both ER-positive and ER-negative tumors that had subsequently metastasized to visceral sites had significantly larger mean maximal diameters than the tumors of the same receptor status that had not undergone detectable distant metastasis (*P* = 0.0001 and 0.004, resp.). Similarly, as a group, the ER-positive tumors that had metastasized to bone were significantly larger than the ER-positive tumors without metastases (*P* = 0.0001); there were too few ER-negative tumors with bone metastases to permit inclusion in the statistical comparisons. Triple-negative tumors that had metastasized to visceral sites had significantly larger diameters than the tumors that had not undergone detectable distant metastasis (4.3 ± 0.5 cm versus 2.8 ± 0.2 cm; *P* = 0.007) but were indistinguishable from the ER-negative tumors that expressed PR and/or HER2 (Table 5).

**Figure 2:**
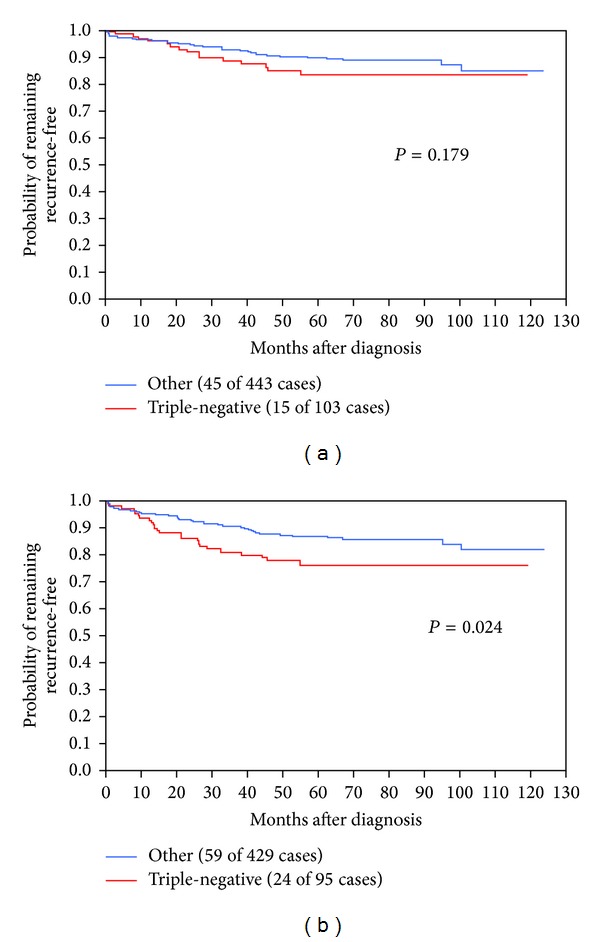
Rate of distant recurrence to bone (a) and to viscera (b) after breast cancer diagnosis in triple-negative women compared with other cancer types.

The probability of developing visceral metastasis within 10 years of diagnosis was significantly higher among the women with triple-negative tumors compared with other forms of breast cancer (Figure 2). The probability of developing a visceral metastasis as the first site of distant recurrence was higher (26%) among the women with triple-negative disease than for women with other subtypes of breast cancer (16%; *P* < 0.01). In contrast, there was no significant difference in the rate of developing bone metastases between the two groups. The probability of developing a bone metastasis within 10 years was similar for women with triple-negative breast cancer (25%) compared with other subtypes (18%; *P* = 0.20).

### 3.4. BMI and Obesity

There were 548 women for whom the BMI was recorded at the time of their initial surgical treatment, and of these 205 (37.4%) had values of 30 or higher and were classified as obese; another 191 (34.9%) were considered to be overweight. There were no significant differences in the mean BMI values, or in the frequency of obesity, in the patients who had not developed bone or visceral metastases during the study period compared with those in the two metastatic groups (Table 1).

We evaluated the relationships between the BMI and the ER and the triple-negative phenotypes. For the statistical comparisons, the lean and overweight women were combined into a single nonobese group (BMI < 30 kg/m^2^). In the nonmetastatic group, neither the mean BMI values nor the prevalence of obesity differed significantly between patients with ER-positive or ER-negative tumors (data not shown). However, these same relationships were significantly different when the comparison was limited to the triple-negative tumors. The mean BMI in kg/m^2^ was 30.1 ± 0.6 in the triple-negative group compared with 28.7 ± 0.3 for non-triple-negative group (*P* = 0.006). We observed that 47.0% of the patients with triple-negative tumors were obese compared with only 35.1% of those in other receptor categories (*P* = 0.031). However, there was no significant difference in the proportion of lean and obese patients with metastatic disease between ER and triple-negative groups. To adjust for possible confounders, a multiple logistic regression was run with metastasis as the response and BMI, age, receptor status, and tumor size as factors in the model. Neither age (*P* = 0.21) nor BMI (*P* = 0.56) was significantly associated with metastases, while receptor status (*P* = 0.0395; triple-negative versus other subtypes) and tumor size (*P* < 0.0001) were associated with spread of the disease.

## 4. Discussion and Conclusion

West Virginia, with a population that is approximately 95% white, does not have an unusual high incidence of breast cancer; indeed, with an average annual age-adjusted figure of 119.2 per 100,000 during the period of the study, it ranked 41st of 46 states and the District of Columbia. However, as is also seen most typically in the African-American segment of the United States female population [12], the low incidence rate is accompanied by a relatively high rate of breast cancer mortality, with West Virginia ranking 16th of the 50 states and the District of Columbia.

Patients who were diagnosed before 1999 were excluded from the present study to avoid the period during which a pronounced increase in the use of screening mammography was taking place, with its shifting influence on breast cancer stage at diagnosis. The result was that, with the new high level of early detection, only 24.9% of the patients had axillary lymph node involvement. This is consistent with a report by Jubelirer et al. [24] that in their study of the changing pathological features of breast cancer associated with screening mammography in West Virginia, the incidence of lymph node positive disease fell from 41% to 28%. An inevitable consequence of this, combined with the low populations of many of the individuals, mostly rural, counties, and the low breast cancer incidence rate, is that the absolute number of patients at risk for distant metastases was small compared with other areas of the United States.

In the present study, the first distant metastases were located at a visceral site in 55.8% of cases compared with 44.2% occurring in bone. This difference was not statistically significant, but most other investigators who studied predominantly or exclusively white women found that the skeleton was the more common site of first distant metastasis, when it was related to longer survival [9, 14–16, 25]. There is uniform agreement that bone as the first distant metastatic site is most commonly associated with ER-positive primary tumors, whereas the majority of visceral metastases arise from ER-negative tumors [25–28]; further, metastasis to visceral sites is related particularly to the triple-negative phenotype [8, 9]. These relationships between metastatic behavior and receptor status were evident in the present study. We performed additional analysis to evaluate whether the metastatic pattern was the same in cases where the receptor status (ER, PR, and HER2/neu expression) was incomplete. Of note, the metastatic patterns of 113 breast cancer cases without complete receptor information were compared with those women whose receptor status was known. We found that the metastatic patterns were comparable. Only 3% of them had visceral metastases compared to 9% that had complete information (*P* = 0.027). No significant difference was found for bone metastases. There were fewer bone metastases among the women with incomplete information. For all metastases, 8% of women with incomplete receptor status had a metastatic event, whereas 15% with a complete receptor history had distant metastases (*P* = 0.048).

Overall, patients with distant metastases were more likely to have lymph node involvement, but there was no particular association with visceral recurrences. This may have been due to small numbers and weak statistical power. Rack et al. [29] found that axillary lymph node involvement at the time of diagnosis was a biomarker of an aggressive phenotype and that the site of first metastasis was predominantly visceral in node positive patients. Moreover, we did find that triple-negative breast cancers that had undergone visceral metastasis and hence were likely to fulfill the prediction of a short survival time had a high prevalence of lymph node involvement.

Earlier [13], we found that breast cancer patients in West Virginia had a frequency of triple-negative tumors that was higher than that generally reported in white American women and which was associated with greater size and higher histologic grade. The large tumors associated with the triple-negative phenotype were more likely to be accompanied by axillary lymph node metastases, but the prevalence of node positive tumors was no higher in patients with triple-negative tumors, a relationship that had been observed previously by some [8, 30] but not all investigators [6].

The Nottingham system for the assessment of histologic grade used here is based on an accurate mitotic count and a semiquantitative evaluation of two morphologic features, the percentage of nuclear pleomorphism, and the degree of tubule formation [31]. As is to be expected from its dependence on the level of mitotic activity, there is a strong positive correlation between tumor grade and the expression of the proliferation marker Ki-67, the high levels of which predict a poor breast cancer prognosis [32]. Likewise, histologic grade is an index of tumor biological aggressiveness and high grade is associated with poor patient outcome [31]. The clinical contribution of grade has become more important relative to lymph node status due to the earlier detection of breast cancer by mammographic screening. This is evident in the present study, where only 28.4% of the patients of known status were node positive, whereas 55.5% of the tumors were grade 3. Moreover, 51.9% of the women with lymph node negative breast cancer had grade 3 tumors. In our earlier study from West Virginia [13], triple-negative tumors were more likely to be of high histologic grade, and here we found that 80.9% of the 89 nonmetastasizing triple-negative tumors were grade 3 compared with 50.6% of all of the tumors that had not yet undergone distant metastasis.

Caution is necessary when comparing studies of breast cancer histologic grade because of variations in the assessment methods and in the reproducibility of results between individual pathologists. Nevertheless, it is noteworthy that in a large multicenter study performed by Chlebowski et al. [33] the histologic grade of tumors from African-American women, who have a relatively poor breast cancer prognosis, was indicative of a higher degree of biological aggression compared with white American women. Also, the high incidence of poorly differentiated, grade 3, tumors (52.5%) and low incidence of well-differentiated, grade 1, tumors (16.2%) in African-American patients were similar to that of the 592 white West Virginian patients in our study (grade 3, 55.6%; grade 1, 12.2%), who represent a state whose predominantly white female population has a relatively poor breast cancer prognosis and a high prevalence of socioeconomic deprivation and obesity.

We reported previously that triple-negative primary breast cancers and obesity are related [13], and others have shown that both obesity [1, 20] and this tumor type [6, 8, 9] have an increased propensity for distant metastasis, in particular, for spread to visceral sites [9, 20]. Although the relationship between obesity and triple-negative tumors was confirmed by Trivers et al. [19] we found no demonstrable associations of distant metastases with obesity in our study group as a whole, or in those with the triple-negative phenotype. It may be that the overall prevalence of obesity in these women was sufficient to provide for an optimal stimulation of body weight-related factors involved in the promotion of distant, visceral, breast cancer metastasis and that successful completion of the process is then determined by unrelated components of the metastatic cascade. In this context, it is noteworthy that of the 548 women in the present study with a record of their BMI at the time of diagnosis, 37.4% were obese; in two large studies from the United States that included both premenopausal and postmenopausal breast cancer patients, only 20.0% of 3,385 and 22.3% of 1,491 women, respectively, had BMI values of 30.0 kg/m^2^ or higher [34, 35]. Wynder and Stellman [36] discussed a similar problem that of the “overexposed” control group, in which there is a narrow range of exposure to a postulated risk factor for cases and controls, in relation to dietary studies in cancer etiology.

The large tumor size with an increased frequency of distant metastases, the dominance of grade 3 tumors even in the absence of axillary lymph node involvement, and its association with visceral metastases, and an unusually high incidence of triple-negative tumors in these white women, are all consistent with the comparatively poor survival of breast cancer patients in West Virginia. It is important to point out that this situation is very likely to be equally applicable to the rest of Appalachia.

The biological mechanisms involved in the relatively poor clinical outcome of breast cancer patients in West Virginia require investigation, but clues are provided by the high prevalence of triple-negative tumors with a propensity for visceral metastasis. Vascular invasion is a critical step in the metastatic process and was reported to be particularly common in triple-negative breast cancer [37, 38] and in obese breast cancer patients [39]. Peritumoral vascular invasion involving the lymphatic and blood vessels adjacent to the tumor mass has been associated with greater tumor size and higher grade and a short metastasis-free interval [40, 41] and reduced recurrence-free and overall survival [41]. Leptin is one of several adipokines secreted from adipose tissue that stimulate breast cancer cell proliferation and invasion and also the angiogenic process that is essential for distant metastasis [42]. Leptin interacts with insulin-like growth factor-1 in triple-negative breast cancer cells to transactivate the epidermal growth factor receptor and so promote tumor cell proliferation, migration, and invasion [43]. In support of the clinical significance of these experimental observations, a polymorphism in the leptin receptor gene with elevations in the serum leptin concentrations was reported in patients with triple-negative breast cancers [44] and high tumor tissue leptin and leptin receptor expression observed in association with distant metastases [45]. Future studies should address whether adipocyte-derived signals such as leptin influence breast cancer tumorigenesis and the pattern of metastatic spread.

In summary, breast cancer patients in rural Appalachia have a high prevalence of obesity and poverty, together with excessive expression of the triple-negative phenotype. The probability of developing visceral metastasis within 10 years of diagnosis was significantly higher among the women with triple-negative tumors compared with other forms of breast cancer, but this relationship was not influenced by increasing body mass index. Metastatic spread to visceral organs in the triple-negative phenotype was combined with the burden of more advanced tumors. Women with breast cancer in other disadvantaged segments of Appalachia may exhibit a triple-negative phenotype that is associated with early, distant metastasis.

## Figures and Tables

**Table 1:** Clinical features and metastatic distribution of patient population.

	No metastases at followup *n* = 592 (%)	Distant metastases		*P* value
		Bone *n* = 42 (%)	Visceral *n* = 53 (%)	
Age at diagnosis*	58.7 ± 0.5^a^	53.6 ± 2.1^b^	56.0 ± 1.9^ab^	0.038
<50	156 (26.3)	17 (40.5)	21 (39.6)	0.028
≥50	436 (73.7)	25 (59.5)	32 (60.4)	
BMI (kg/m^2^)	28.8 ± 0.3	29.9 ± 1.1	28.7 ± 0.9	0.642
<25	131 (27.4)	5 (15.2)	16 (35.6)	0.351
25–29.9	164 (35.1)	14 (42.4)	13 (28.8)	
≥30	175 (37.5)	14 (42.4)	16 (35.6)	
Missing data	122	9	8	
Tumor size (cm)	2.2 ± 0.8^b^	3.6 ± 0.3^a^	4.1 ± 0.3^a^	<0.0001
T1 <2	292 (52.5)	10 (29.4)	8 (17.8)	<0.0001
T2 2–5	222 (39.9)	13 (38.2)	18 (40.0)	
T3 >5	42 (7.6)	11 (32.4)	19 (42.2)	
Missing data	36	8	8	
Tumor grade				
1	73 (14.2)	1 (2.5)	3 (6.0)	0.0002
2	175 (34.1)	12 (30.0)	7 (14.0)	
3	265 (51.7)	27 (67.5)	40 (80.0)	
Missing data	79	2	3	
Lymph node				
(i) Positive^†^	124 (21.5)	16 (39.0)	27 (50.9)	<0.0001
(ii) Negative	453 (78.5)	25 (61.0)	26 (49.1)	

*Means are presented with standard errors.

^†^Lymph node status not available in 16 cases.

^a,b^Means without the same superscript are significantly different.

**Table 2:** First site of distant metastases and median disease free interval in 95 patients.

Anatomic site	*n* (%)	Median disease free interval, months (CI)	*P* value
Bone	42 (44.2)	19.9 (3.2–27.5)	0.079^†^
Viscera	53 (55.8)	13.9 (9.1–20.3)	
Liver*	21/53 (39.6)	20.9 (6.4–26.4)	<0.024^‡^
Lung*	20/53 (37.7)	9.6 (2.3–14.1)	
Brain	11/53 (20.8)	25.8 (8.1–40.1)	
Ovary	1/53 (1.9)	13.3	

*There were 8 patients who presented simultaneously with liver and lung metastases; according to protocol, liver took precedence when assigning first metastatic site.

^†^ *P* represents a comparison between bone and combined visceral sites using log-rank test.

^‡^ *P* represents a comparison within visceral sites excluding the ovary using log-rank test.

CI denotes lower and upper 95% confidence intervals.

**Table 3:** Pattern of metastatic spread for tumors with known steroid receptor status and HER2 overexpression.

Category	Number of cases	ER- positive	PR- positive	HER2 overexpression*	Triple- negative
No metastases	488	345 (70.7)	316 (64.7)	98 (20.1)	107 (21.9)
Metastases					
Bone	42	33 (78.6)	29 (69.0)	5 (13.9)	6 (14.3)
Viscera	53	22 (41.5)	18 (33.9)	6 (12.0)	22 (41.5)
No metastases versus mestastases		0.0005	0.0004	0.245	0.002
Bone versus viscera		0.0003	0.0008	0.796	0.003

*HER2 expression was unknown in 10 cases.

**Table 4:** Patterns of metastatic spread by lymph node status and tumor grade in women with triple-negative and other breast cancers.

	No metastases at followup	Distant metastatic sites	
		Bone	Visceral
Triple-negative (TN)			
Lymph node^a^			
(i) Positive	25 (24.3)	3 (50.0)	15 (68.2)
(ii) Negative	78 (75.7)	3 (50.0)	7 (31.8)
Other breast cancers			
Lymph node			
(i) Positive	99 (20.9)	14 (38.9)	12 (38.7)
(ii) Negative	375 (79.1)	22 (61.1)	19 (61.3)
*P* value	0.610	0.454	0.033

Triple-negative			
Tumor grade^b^			
1	7 (6.5)	0	0
2	20 (18.7)	2 (33.3)	2 (9.1)
3	80 (74.8)	4 (66.7)	20 (90.9)
Other breast cancers			
Tumor grade			
1	66 (16.3)	1 (2.9)	3 (10.7)
2	155 (38.2)	10 (29.4)	5 (17.9)
3	185 (45.5)	23 (67.7)	20 (71.4)
*P* value	<0.0001	0.839	0.092

^a^Nodal status not tested in 16 cases.

^b^Tumor grading unavailable in 95 cases.

**Table 5:** Tumor size in relation to ER and triple-negative status^†^.

Category	ER-positive	ER-negative	Triple-negative	ER-negative Not triple-negative
No metastases	2.0 ± 0.9 (389)^b^	2.7 ± 0.2 (150)^b^	2.8 ± 0.2 (103)^b^	2.8 ± 2.0 (47)
Metastases				
Bone	4.2 ± 0.3 (26)^a^	1.6 ± 0.7 (8)^b^	1.4 ± 0.9 (5)^b^	—
Visceral	3.9 ± 0.4 (18)^a^	4.1 ± 0.4 (26)^a^	4.3 ± 0.5 (19)^a^	3.7 ± 1.5 (8)
*P* value	<0.0001	0.001	0.007	

^†^Tumor size in centimeters, cm ± standard error; (*n*): number of cases.

^a,b^Means without the same superscript are significantly different.
